# Effect of C/N Ratio and Media Optimization through Response Surface Methodology on Simultaneous Productions of Intra- and Extracellular Inulinase and Invertase from *Aspergillus niger* ATCC 20611

**DOI:** 10.1155/2013/508968

**Published:** 2013-09-15

**Authors:** Mojdeh Dinarvand, Malahat Rezaee, Malihe Masomian, Seyed Davoud Jazayeri, Mohsen Zareian, Sahar Abbasi, Arbakariya B. Ariff

**Affiliations:** ^1^Department of Microbiology, Faculty of Biotechnology and Biomolecular Sciences, Universiti Putra Malaysia, 43400 Serdang, Selangor, Malaysia; ^2^Institute of Bioscience, Universiti Putra Malaysia, 43400 Serdang, Selangor, Malaysia; ^3^Department of Food Science, Faculty of Food Science and Technology, Universiti Putra Malaysia, 43400 Serdang, Selangor, Malaysia; ^4^Department of Bioprocess Technology, Faculty of Biotechnology and Biomolecular Sciences, Universiti Putra Malaysia, 43400 Serdang, Selangor, Malaysia

## Abstract

The study is to identify the extraction of intracellular inulinase (exo- and endoinulinase) and invertase as well as optimization medium composition for maximum productions of intra- and extracellular enzymes from *Aspergillus niger* ATCC 20611. From two different methods for extraction of intracellular enzymes, ultrasonic method was found more effective. Response surface methodology (RSM) with a five-variable and three-level central composite design (CCD) was employed to optimize the medium composition. The effect of five main reaction parameters including sucrose, yeast extract, NaNO_3_, Zn^+2^, and Triton X-100 on the production of enzymes was analyzed. A modified quadratic model was fitted to the data with a coefficient of determination (*R* ^2^) more than 0.90 for all responses. The intra-extracellular inulinase and invertase productions increased in the range from 16 to 8.4 times in the optimized medium (10% (w/v) sucrose, 2.5% (w/v) yeast extract, 2% (w/v) NaNO_3_, 1.5 mM (v/v) Zn^+2^, and 1% (v/v) Triton X-100) by RSM and from around 1.2 to 1.3 times greater than in the medium optimized by one-factor-at-a-time, respectively. The results of bioprocesses optimization can be useful in the scale-up fermentation and food industry.

## 1. Introduction

In *A. niger* ATCC 20611, there are two associated intra- and extracellular glycoproteins enzymes to target the *β*-2, 1 linkage: inulinase (EC 3.2.1.7) and invertase (EC 3.2.1.26) [1, 2]. These enzymes exhibit corresponding hydrolytic activities towards sucrose but differ in their specificities for higher-molecular-weight oligosaccharides and fructans of the inulin type [3]. The potential of microorganisms to produce enzymes is vastly exploited for industrial purposes.* A. niger* ATCC 20611 efficiently utilizes wide a variety of inexpensive nutritional compounds to produce several intra- and extracellular enzymes. The productivity of many fermentation processes is affected by different parameters such as nutritional (medium composition) and physicochemical (agitation rates, pH value, inoculum size, and temperature) conditions to which the microorganism is exposed. The optimal design of culture media is an important aspect to be considered in the development of a fermentation process. The formulation of media containing complex nutrients is generally preferred for large-scale fermentations, since it leads to the development of cost-effective processes that support maximum product yield. In the initial formulation of the medium in batch culture, an effort is made to understand the best source of carbon and energy, as well as the regulatory aspects of the enzyme. RSM is a mathematical modelling system which assesses the relationships between the response(s) and the independent variables [4] and defines the importance of the independent variables, alone or in combination, in the model. In addition, RSM has been furnished by ANOVA analysis, which can help to statistically analyze the whole model produced, every single parameter involved and its interactions [5, 6]. 

The production of enzymes through experimental design has gained much attention in biotechnology and industry studies for producing inulinase and invertase. Also, the selection of suitable microorganism is an important aspect of experimental design for producing the enzymes. *A. niger* is important in the food, beverage, and pharmaceutical industries and known as a potential producer of enzymes and organic acids [7, 8]. Furthermore, it is generally regarded as safe (GRAS) status, indicating that enzymes and other products produced by *A. niger* are deemed safe to be used for pharmaceutical and food applications [9]. Generally, the catalytic activities of inulinase (I) and invertase (S) are described in terms of I/S ratio (relative activities with inulin and sucrose) and employed to distinguish between inulinase and invertase [10]. Differences in I/S ratios and *K*_*m*_ value were considered by some authors to be insufficient for distinction and characterization of the enzymatic complex. When I/S ratio is higher than 10^−2^, the inulinase production is preponderated in the culture, while for invertase production, the I/S ratio lower than 10^−4^ indicted the higher production [11]. A low I/S ratio (high activity with sucrose) is taken to indicate invertase. The naming of inulinase or invertase as *β*-fructosidase is based on their relative hydrolytic capacity for inulin and sucrose (I/S) [12]. 

Therefore, the main objective of the present study is to improve simultaneous productions of intra- and extracellular inulinase (exo- and endo) and invertase by *A. niger* ATCC 20611 through the optimization of the medium by RSM. At first, the effects of nutritional factors on intra- and extracellular inulinase and invertase productions were investigated by one-factor-at-a-time. Then, the concentration of the medium components was optimized using RSM. The main advantage of this study is the achievement of the higher productivities by using low cost carbon source and energy requirements. Based on our knowledge, optimization of two intra- and extracellular glycoproteins production by RSM has not yet been reported. 

## 2. Materials and Methods

### 2.1. Microorganism, Preparation of Inoculum, and Medium Composition

*A. niger* ATCC 20611 was obtained from the American Type Culture Collection (ATCC), Rockville, Maryland, USA, and was used in this study for intra- and extracellular inulinase and invertase productions. The strain was maintained at 4°C on potato dextrose agar (PDA) and subcultured every 3 weeks. The spores were harvested and suspended in sterile distilled water containing 0.01% (v/v) Tween 80 to obtain approximately 2.0 × 10^6^ spores/mL. Preliminary experiments were performed by using a basal medium containing inulin 1% (w/v) and peptone 0.5% (w/v). The initial pH of the basal medium was adjusted at 6.5, prior to sterilization at 121°C for 15 min. The basal medium was inoculated with a 6% (v/v) of stock culture and incubated at 30°C with 150 rpm shaking for 96 h [13, 14]. All the fermentations procedures were carried out in triplicate in 250 mL Erlenmeyer flasks containing 50 mL of the basal medium. 

### 2.2. Inulinase and Invertase Production Media

Six different media for extracellular inulinase and invertase productions were tested for the production of the enzymes by *A. niger* ATCC 20611. The composition of the media included the medium A (g/L): yeast extract (3 g), malt extract (3 g), peptone (5 g), and dextrose (10 g) [15]; medium B (g/L): potato dextrose broth (40 g); medium C (%): for the production of inulinase (inulin 1% (w/v), NH_4_Cl 2.4% (w/v), and Mg_2_SO_4_·7H_2_O 1.2% (w/v)) and for invertase production (inulin 5% (w/v), NH_4_Cl 4.8% (w/v), and Mg_2_SO_4_·7H_2_O 1.2% (w/v)) [2]; medium D (g/L): sucrose (20 g), yeast extract (2 g), NaNO_3_ (2 g), MgSO_4_·7H_2_O (0.05 g), and K_2_HPO_4_ (0.5 g) [16]; medium E (g/L): inulin (10 g), yeast extract (1.5 g), NH_4_NO_3_ (2.3 g), (NH_4_)_2_HPO_4_ (3.7 g), KH_2_PO_4_ (1.0 g), and MgSO_4_ (0.5 g) [17]; medium F (g/L): inulin (2 g), peptone (2 g), (NH_4_)H_2_PO_4_ (1.2 g), NaCl (0.5 g), and MgSO_4_·7H_2_O (0.05 g) [18]. 

### 2.3. Experimental Design

RSM offers a large amount of information from a small number of experiments because of using special designs that help the appropriate model to be fitted to the response(s). The experiments were generated by central composite design (CCD). All treatment combinations were performed in 250 mL Erlenmeyer flasks containing 50 mL of the medium which had been optimized by one-factor-at-a-time method [13, 14]. After 96 h of incubation, each flask was assayed for enzymes activity. RSM modelling was used to optimize the best range of the five effective nutrient factors (sucrose, yeast extract, NaNO_3_, Zn^2+^, and Triton X-100), which resulted from previous studies [13, 14], on the responses of intra- and extracellular inulinase and invertase productions, biomass, and C/N ratio. The lowest and highest concentrations of the selected ingredients in the media were sucrose (4% to 16%, w/v), yeast extract (1% to 4%, w/v), NaNO_3_ (1% to 3%, w/v), Zn^2+^ (1 mm to 2 mm, v/v), and Triton X-100 (0.5% to 1.5%, v/v). The independent variables and their levels are shown in Table 1. The CCD design experimental data were employed using Design Expert version 6.06 (Stat-Ease Inc. Minneapolis, USA) and then interpreted. By using a five-factor and three-level CCD, 26 treatment combinations were generated as small type in experimental design. Often four or more replicates at center point are considered to estimate the experimental error (pure error variance). This offered an adequate estimate of the variation of the response and provided the number of degrees of freedom needed for an adequate statistical test of the model [5, 6, 19]. Behaviour of the system is explained by the following second-order polynomial equation:

(1) $$\begin{array}{c} Y=\beta_{0}+\sum_{}^{}\;\beta_{i}x_{i}+\sum_{}^{}\;\beta_{ii}x_{i}^{2}+\sum_{}^{}\;\beta_{ij}x_{i}x_{j}, \end{array}$$

where *Y* is the predicted intra- and extracellular inulinase and invertase productions (U/mL), biomass (mg/mL), and C/N ratio; *x*_*i*_ and *x*_*j*_ are the parameters (sucrose, yeast extract, NaNO_3_, Zn^+2^, and Triton X-100); *β*_0_ is the intercept term; *β*_*i*_, *β*_*ii*_, and *β*_*ij*_ are the linear, squared and interaction coefficients, respectively. In order to test the estimation capabilities of the technique, the predicted responses obtained from RSM were compared with the actual responses. The *R* ^2^ was determined for finding the best models by measuring the amount of the reduction in the variability of response which represses variables in the model and must be close to 1. 

### 2.4. Analytical Method for Intra- and Extracellular Inulinase and Invertase Activities

The supernatants of the cultures at 96 h of fermentation were harvested by centrifugation at 4°C (20 min, 10,000 ×g) in triplicate to determinate extracellular inulinase activity (I) and invertase activity (S). Then, cell pellet was used as a source of intracellular inulinase and invertase activities. For the extraction of intracellular enzymes, two methods were used. In the first method, the pellet was resuspended in 50 mL of sodium acetate buffer (200 mM, pH 5.0) with vigorous vortexing followed by incubation at 30°C and 150 rpm for 30 min. Then, it was sonicated on ice in glass tubes using a Branson Sonic Power Sonicator (48 Bransonic Power, 40 W, 30 s with 30 s cooling periods) for 5 min followed by centrifugation at 4°C (20 min, 10,000 ×g). In the second method, the mycelia were blotted with filter paper and grounded in a porcelain mortar with sand at 4°C and then resuspended in 50 mL of sodium acetate buffer (200 mM, pH 5.0) [20]. Cell debris was removed by centrifugation at 4°C (20 min, 10,000 ×g). The supernatant was used as the crude intracellular inulinase and invertase enzymes. Inulinase and invertase assay was carried out according to Dinarvand et al. [13, 14]. Briefly, 0.5 mL of 1% (w/v) substrate (inulin and sucrose) in sodium acetate buffer (200 mM, pH 5.0) was added to 0.5 mL of supernatant containing crude inulinase and invertase. Then, the reaction was incubated at temperature of 50°C for 20 min. The same reaction mixture without the enzyme extract was used as the control. The amount of the reducing sugars in the reaction mixture was assayed using the DNS method [21]. Inulinase and invertase activities (U/mL) were defined as 1 *μ*mol of fructose and glucose liberated per min under the assay conditions, respectively. Specific activity was defined as a rate of total enzyme activity over protein content in milligram. Experiments were carried out in triplicate runs, and all standard deviations were lower than 10%.

### 2.5. Determination of Protein, Biomass, Polysaccharide, and Nitrogen Content

Total protein was determined according to Bradford [22], using bovine serum albumin (BSA) as standard. The biomass was determined by dry weight measurement [13, 14]. The polysaccharide content was determined using the phenol-sulphuric acid method as described by Dubois et al. [23]. The nitrogen content in the medium was determined using the Kjeldahl method [24]. All the assays were carried out in triplicate. 

## 3. Results and Discussion

### 3.1. Effect of Different Media on Inulinase and Invertase Productions

The ability of *A. niger* ATCC 20611 to simultaneously produce inulinase and invertase was studied in six different liquid media. The results (Table 2) indicated that all the media supported inulinase and invertase productions, although the levels of inulinase and invertase productions were shown to be greatly different. Maximum productions of inulinase (2553 U/mL) and invertase (1983 U/mL) in medium D were significantly higher than other production media containing sucrose as carbon source, yeast extract and NaNO_3_ as nitrogen sources, and Mg^+2^ and K^+2^ as metal ions. The lowest production of inulinase (157 U/mL) and invertase (536 U/mL) in medium A were observed with the composition of dextrose as carbon source, yeast extract, malt extract, and peptone without any metal ions. The productions of inulinase and invertase are mostly inducer dependent. The different media have various effects on inulinase and invertase productions based on the physiological and biochemical pathways of the fungi. The higher inulinase and invertase productions obtained in fermentation by various microorganisms such as *A. japonicas*, *A. ficuum, Candida guilliermondii* TISTR 5844, and *A. niveus* Blochwitz 4128URM ranged from 0.5 to 810 U/mL [2, 16, 18, 19]. Based on the previous-obtained results, inulinase and invertase productions by *A. niger* ATCC 20611 were 10 fold higher than other microorganisms, suggesting that this strain could be a potential source for industrial section.

### 3.2. Comparative Evaluation of Extraction Methods for Intracellular Enzymes

In order to monitor the cell disruption process, two different methods of mechanical cellular breakage, sonication and porcelain mortar, were tested. Optimized conditions of sonication and porcelain mortar presented comparable yields of enzyme activity (Table 3). The maximum intracellular inulinase (158 U/mL) and invertase (295 U/mL) activities were obtained by using the sonication method and the porcelain mortar method which showed 82 and 127 U/mL activities, respectively. In order to obtain the optimal method for extracting intracellular inulinase and invertase, we aimed at establishing sonication as the best method for enzymes extraction in this study. Secreted invertase resides mainly in the cell wall as an octamer. The equal amount of invertase present in the culture fluid as well as the fraction, which is removable from the cells by treatment with sonication, was found to be composed of dimers. It has been suggested that oligomerization helped to retain the enzyme within the cell wall [3]. Similarly, the inulinase of yeast was associated with the cell wall. In contrast to the invertase, much more of the inulinase from fungi was actually secreted into the culture fluid. When *A. niger* ATCC 20611 was grown under conditions which derepress the enzyme production, around 80% of its inulinase was secreted into the culture fluid and the rest of the enzyme was gained by sonication from the cell wall. In comparison to the biochemistry of the invertase of fungi, less information has been reported about the biochemistry of inulinase. 

### 3.3. Effect of Time on the Intra- and Extracellular Inulinase and Invertase Productions

The *A. niger* ATCC 20611 strain was grown in the basal medium; biomass, intra- and extracellular inulinase and invertase productions, and pH were measured during 168 h incubation at different time points. The maximum intra- and extracellular inulinase and invertase productions and biomass were obtained after 96 h of cultivation at 30°C in a shaker incubator with agitation speed of 150 rpm. Intra- and extracellular inulinase and invertase productions were shown to coincide with the exponential growth phase. There was a reduction in pH from 6.5 to 3.0 during intra- and extracellular inulinase and invertase productions, and maximum enzymes production was observed at pH 4.6 (data not shown). The maximum productions of inulinase and invertase were obtained at the end of the logarithmic growth phase [20, 25]. It was an interest to determine inulinase production together with invertase; many microbial preparations of inulinase possess remarkable invertase activity (S) accompanying the inulinase activity (I), where their catalytic activity is described in terms of I/S (Inulin/Sucrose) ratio [25, 26]. There was a significant positive correlation (*r*) coefficient at *P* ≤ 0.01 between growth on one side and the production of inulinase (*r* = 0.98) and invertase (*r* = 0.99) on the other side. In addition, inulinase and invertase were also positively correlated (*r* = 1). The enzymes production by *A. niger* ATCC 20611 was growth associated, because the maximum activities were obtained at the stationary phase [26]. Higher inulinase and invertase productions in a very short fermentation time could be an advantage of using *A*. *niger* ATCC 20611. Increasing incubation time led to reduction in enzymes production. Reduced enzymes activities after 120 h of fermentation could be either due to the decrease in nutrient availability in the medium, proteolytic activity, or catabolic repression of enzymes [26, 27]. Reduction in the culture pH may have also made an impact on enzymes production [26]. The decrease in pH was probably due to the deamination of some amino acids or formation of organic acids [13, 14]. 

### 3.4. Response Surface Methodology Study on Optimization of Medium Ingredients for Intra- and Extracellular Inulinase and Invertase Productions and Biomass

#### 3.4.1. Model Fitting and Analysis of Variance (ANOVA)

The average intra- and extracellular inulinase and invertaseproductions, biomass, and C/N ratio were obtained after 4 days of fermentation in 26 experiments of the chosen experimental design. The best fitting models were determined through quadratic regressions. Finally, the modified quadratic model was highly significant (*P* < 0.001) to represent the actual relationships between the responses and the significant variables. Analysis of variance (ANONA) was used to evaluate the significance of the coefficients of the modified quadratic model. In the model for all responses, very small “model *P* values” (<0.0002) and large “lack of fit *P* values” (<0.1701) with a suitable *R* ^2^ (≥0.90) and adjusted coefficient of determination was absorbed (Table 4). The second-order regression equation provided the levels of extracellular inulinase and invertase productions, biomass, and C/N ratio as the functions of sucrose, yeast extract, NaNO_3_, Zn^+2^, and Triton X-100. They can be presented in terms of coded factors as in the following equations:

(2) extracellular  inulinase  activity  (U/mL) =+2681.56+88.31A−198.16B−66.73C  +490.15D+268.81E−1057.79A2  −462.17B2−434.03C2+472.26D2  −162.67E2+350.71AB+425.54AC⁡  +290.77BC−330.10DE,extracellular  invertase  activity  (U/mL)   =+2887.82+226.53A+16.96B  +60.23C+440.70D+36.42E  −1057.62A2−399.88B2−403.29C2  +392.21D2−180.19E2+239.56AC⁡  +249.02AE+121.82BE+203.35CE,Biomass  (mg/mL) =+48.30+2.08A+3.93B+0.36C    +11.94D+0.38E−12.79A2−7.72B2  −7.90C2+10.43D2−3.51E2+10.46AC⁡  +9.88AE−8.71BD+9.63CE,CN=+0.61−0.13A+0.021B+0.016C −0.37D−0.027E+0.87A2 +0.42B2+0.42C2−0.23D2 +0.015E2−0.30AC⁡−0.26AE +0.17BD−0.26CE,

where A, B, C, D, and E are sucrose, yeast extract, NaNO_3_, Zn^+2^, and Triton X-100, respectively. ANOVA for the response surface is shown in Table 4. The *R* ^2^ for the quadratic regression model of extracellular inulinase and invertase productions, biomass, and C/N ratio is more than 0.90, and the model was highly significant (*P* < 0.001) for all responses. 

#### 3.4.2. Main Effects and Interactions between Parameters

**Figure 1:**
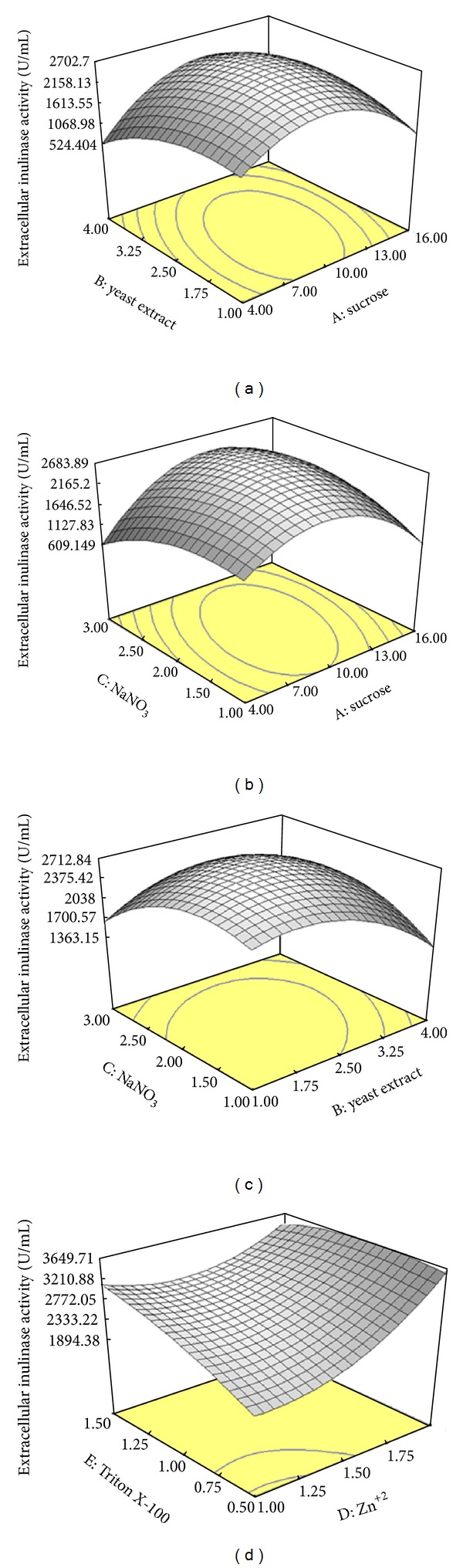
Response surface showing the interaction between five parameters and extracellular inulinase production (U/mL); (a) sucrose and yeast extract, (b) sucrose and NaNO_3_, (c) yeast extract and NaNO_3_, and (d) Zn^+2^ and Triton X-100. Other variables are constant at their center points. The numbers inside the contour plots indicate conversion yield (U/mL) of the extracellular inulinase.

**Figure 2:**
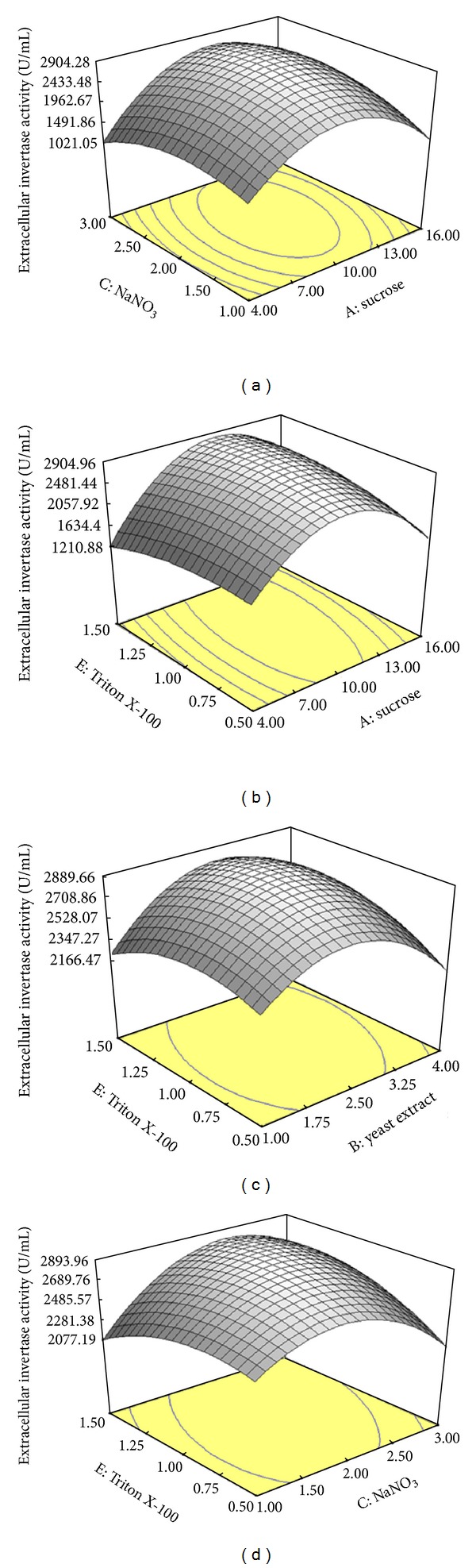
Response surface showing the interaction between five parameters and extracellular invertase production (U/mL); (a) sucrose and NaNO_3_, (b) sucrose and Triton X-100, (c) yeast extract and Triton X-100, and (d) NaNO_3_ and Triton X-100. Other variables are constant at their center points. The numbers inside the contour plots indicate conversion yield (U/mL) of the extracellular invertase.

**Figure 3:**
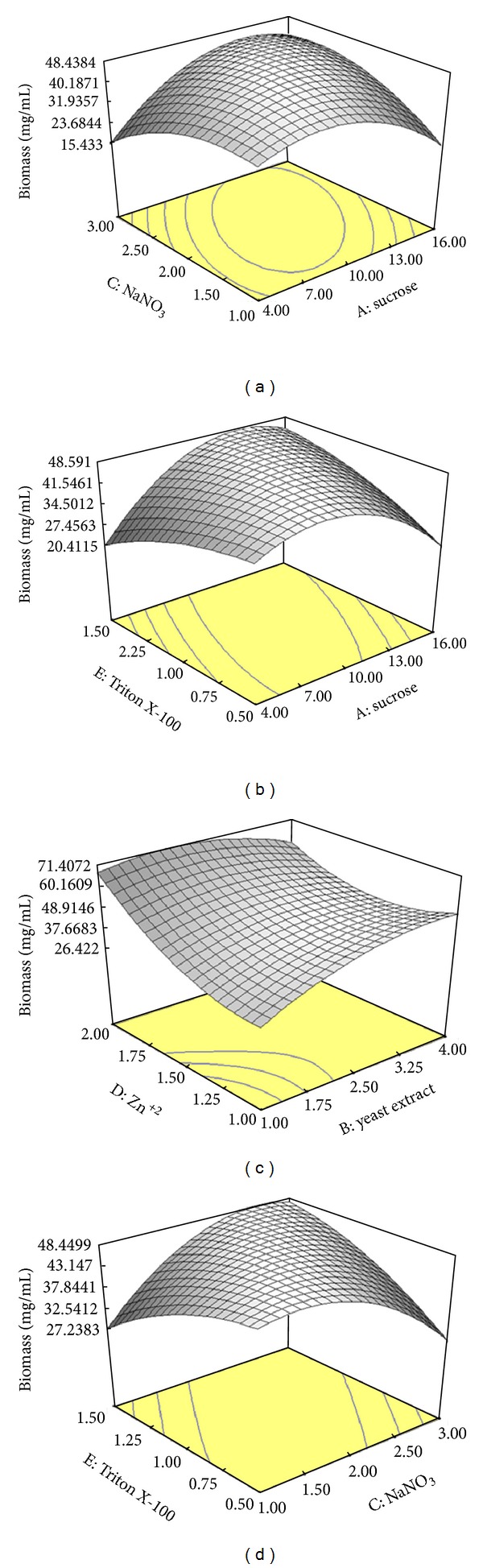
Response surface showing the interaction between five parameters and biomass (mg/mL); (a) sucrose and NaNO_3_, (b) sucrose and Triton X-100, (c) yeast extract and Zn^+2^, (d) NaNO_3_ and Triton X-100. Other variables are constant at their center points. The numbers inside the contour plots indicate conversion yield (mg/mL) of the biomass.

In this research, we tried to analyze, model, and interpret the experimental data using RSM as a mathematical modelling system. The concentration of parameters (sucrose, yeast extract, NaNO_3_, Zn^+2^, and Triton X-100) and the effect of their interactions on intra- and extracellular inulinase and invertaseproductions, biomass, and C/N ratio were determined by CCD of RSM. The interaction effects of the extracellular inulinase and invertase productions, biomass, C/N ratio, and optimal value of variables are clearly represented in the three dimensional response surface plots. Figures 1, 2, and 3 represent the three dimensional plots as functions of extracellular inulinase and invertase productions and biomass, respectively, when other parameters were kept constant at the center point. The results were analyzed via ANOVA. Twenty-six experiments were performed at the different combinations of the factors shown in Table 5. The maximum intra- and extracellular inulinase and invertase productions were obtained with sucrose (10%, w/v) as the sole carbon source (Figures 1 and 2). The composition of the culture medium is the most important factor with influence on enzyme production, growth, and physiology of the cell, and it enhances formation of bioproducts. Readily metabolized or utilized carbon sources in the medium could increase or inhibit the enzyme synthesis. It has been reported that most fungal and yeast strains produce inulinase and invertase in the presence of sucrose, while presence of polysaccharide sugars had an inhibitory effect on both enzymes production [13, 14]. Inulinase and invertase were retained by the cell wall and secreted from cells, residing mainly in the cell wall, where the diffused sucrose can be easily hydrolyzed. Such specific localization of inulinase and invertase may be ecologically beneficial for the efficient scavenging of hydrolyzed products. However, this may not be the case for the other carbon sources because other sugars molecules can hardly penetrate the cell wall and must therefore be hydrolyzed outside the cell wall [13, 14]. Among the carbon sources tested, only sucrose possessed a structure consisting of *β*-1,2 linkage which can be cleared by inulinase and invertase. In addition, the localization of an enzyme, its mode of action, and yield depend upon the kind of microorganism and the substrate used during fermentation [28]. The localization of inulinase and invertase in *A. niger* ATCC 20611 may be different or altered by cultivation conditions. Inulinase and invertase secreted from *A. niger* ATCC 20611 cells resides mainly in the cell wall to perform their physiological function, the cleavage of sucrose molecule diffusible into the cell wall [15, 29]. Such specific localization of inulinase and invertase may be ecologically beneficial for efficient scavenging of hydrolyzed products. The cell wall retention of inulinase and invertase may be advantageous for sucrose utilization in *A. niger* ATCC 20611. The polysaccharides such as inulin are too large to enter the cell wall. Generally, those sugar molecules hardly penetrate into the cell wall, and thus, their hydrolysis occurs outside the cell wall [29]. Therefore, it was suggested that sucrose might act as an inducer for intra- and extracellular inulinase and invertase productions. As lower cost of raw materials for the industrial fermentation processes is mostly preferable, sucrose was selected as the best and most economic of carbon source for intra- and extracellular inulinase and invertase productions by *A*. *niger* ATCC 20611 [10, 12].

Nitrogen sources that produced maximum intracellular inulinase and invertase (data not shown) and extracellular inulinase and invertase (Figures 1 and 2) were obtained by using a mixture of yeast extract (2.5%, w/v) and NaNO_3_ (2%, w/v). Thus, yeast extract and NaNO_3_ were selected as the best sources of nitrogen. In addition, the total specific activity of the enzymes by using the mixture of nitrogen sources (organic and inorganic) was higher compared to using optimum concentrations of organic and inorganic nitrogen sources individually (data not shown). The enzyme productions in fermentation by using a mixture of yeast extract and NaNO_3_ were improved by about 2.5 to 1.6 times, respectively, compared to using yeast extract and NaNO_3_ alone. Enhancement of inulinase and invertase productions in fermentation by using a mixture of yeast extract or NaNO_3_ has also been reported by Skowronek and Fiedurek [31] and Kaur and Sharma [30]. Furthermore, complex nitrogen sources (yeast extract and NaNO_3_) at higher concentrations might have a toxic effect on enzymes production [13, 14].

As shown in Figures 1 and 2, maximum extracellular enzymes productions were observed in the production medium containing Zn^2+^ (1.5 mM) as a trace element followed by Ca^2+^ which enhanced the productions to 77% and 97%, respectively. The same trend was observed for intracellular enzymes (data not shown). Trace elements have significant effects on growth, metabolism, and enzyme synthesis by many microorganisms [15]. Trace metals function as cofactors in enzymatic reactions and also play an important role in enzyme structure stabilization. Metal ions are important in maintaining cell wall rigidity, stabilizing oligomeric proteins, and covalently bounding protein peptidoglycan complexes in the outer membrane [32]. In addition, transition metal ions might change the enzyme activity. This phenomenon was due to the interaction between ions and the enzyme surface charge which could markedly affect the ionization of some amino acid residues, then change the enzyme conformation, and alter the enzyme activity. Zn^2+^ is a multifunctional element found in almost 300 enzymes. It plays essential roles in enzyme production in many microbial species [15, 33]. 

Among the various surfactants supplemented in the production medium, only Triton X-100 (1%, v/v) enhanced intracellular enzymes production (data not shown) and extracellular inulinase and invertase productions (Figures 1 and 2). Triton X-100 in high concentrations decreased the intra- and extracellular enzymes production and biomass of *A*. *niger* ATCC 20611 (data not shown). The studies claim that surfactants affect the cell membrane permeability [25], might affect cell bound enzymes, and enhance the release of enzymes into the medium [34]. The solubilization of membrane bound proteins and phospholipids by Triton X-100 at higher concentrations causes lethal effects on yeast cells [25]. The previous results showed that intra- and extracellular inulinase and invertase productions and biomass were increased with increase in sucrose, yeast extract, NaNO_3_, Zn^+2^, and Triton X-100 up to 10% (w/v), 2.5% (w/v), 2% (w/v), 1.5 mM, and 1% (v/v), respectively. Thereafter, intra- and extracellular inulinase and invertase productions and biomass were decreased with further increase in the parameters. The same trend was observed similar to the other variables (data not shown). The previous model can be used to predict the enzymes production within the limits of the experimental factors. 

#### 3.4.3. Model Validation and Optimum Conditions

From the previous results, the economically optimized composition of fermentation medium for the maximum productions of intra- and extracellular inulinase and invertaseand biomass was as follows: sucrose 10% (w/v), yeast extract 2.5% (w/v), NaNO_3_ 2% (w/v), Zn^+2^ 1.5 mM, and Triton X-100 1% (v/v). Validation of the experimental model was tested by carrying out the batch experiment under optimal operation conditions. The intra- and extracellular inulinase and invertase productions and biomass obtained from experiments were very close to the actual responses predicted by the regression model, which proved the validity of the model. The maximum values for the three responses (extracellular inulinase, extracellular invertase, and biomass) predicted from the model were 3681.21 U/mL, 3854.48 U/mL, and 73.39 mg/mL, respectively. Repeated experiments were performed to verify the predicted optimum conditions. At these optimized conditions, the maximum extracellular inulinase, extracellular invertase, and biomass were found to be 3687.93 U/mL, 3876.72 U/mL responds, and 70.82 mg/mL, respectively (Table 5). The average results from the three replications were coincident with the predicted values and the model was proven to be adequate.  Table 6 presents the optimal combination of parameters that can be used to obtain high enzymes production and biomass. The optimum conditions can be used for future scale-up productions of the intra- and extracellular inulinase and invertase. 

### 3.5. Effect of C/N Ratio on Variables

**Figure 4:**
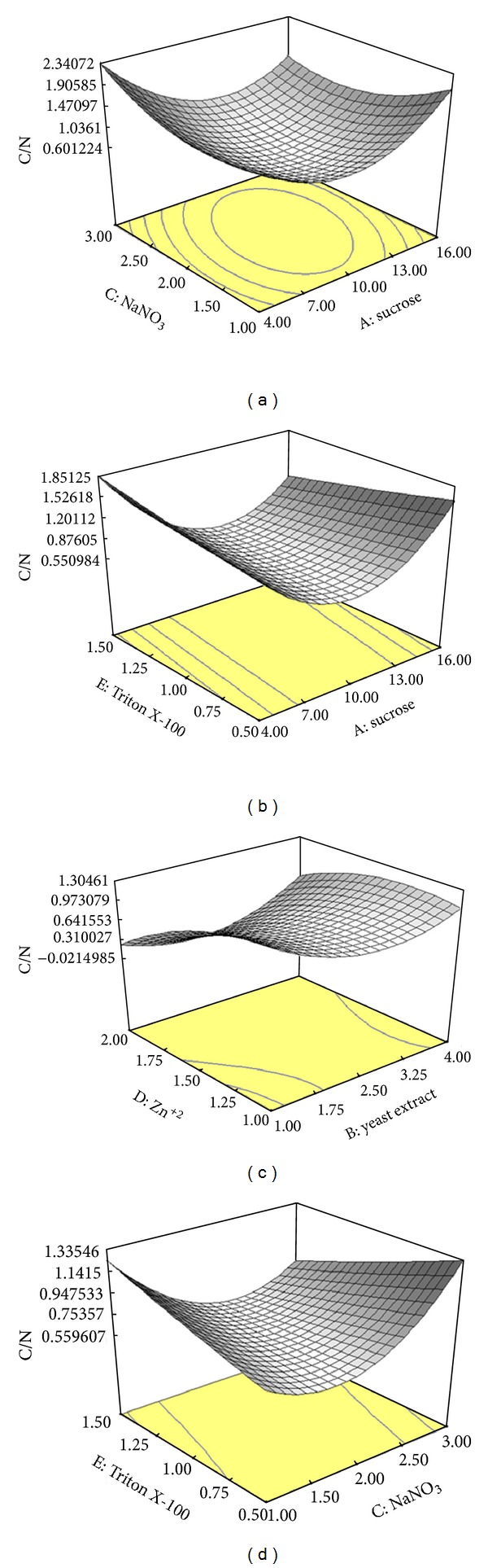
Response surface showing the interaction between five parameters and C/N; (a) sucrose and NaNO_3_, (b) sucrose and Triton X-100, (c) yeast extract and Zn^+2^, and (d) NaNO_3_ and Triton X-100. Other variables are constant at their center points. The numbers inside the contour plots indicate the C/N.

The effect of independent variables on C/N was shown in Figure 4. The results showed the desired C/N ratio obtained in the presence of 10% (w/v) sucrose, 2.5% (w/v) yeast extract, and 2% (w/v) NaNO_3_. Relatively high specific activities of intracellular inulinase (33.71 U/mg), intracellular invertase (43.78 U/mg), extracellular inulinase (73.48 U/mg) and extracellular invertase (76.94 U/mL) were observed in optimized culture with high biomass (73.39 mg/mL), and lower C/N ratios (0.1) (data not shown). In the lowest intra- and extracellular enzymes production, C/N ratio was 2.0. Similar compositions of medium were used to attain high intra- and extracellular enzymes production and growth by *A. niger* ATCC 20611. During media optimization, with increase in the productions of intra- and extracellular inulinase and invertase, the C/N ratio contrarily decreased from 2.6 to 0.1 (Table 5). Similarly, the biomass progressively increased simultaneously with decreasing C/N ratio from 2.6 to 0.1. The highest I/S ratio (1.03) was observed in the medium with a C/N ratio of 0.3. The ratio of I/S sharply increased for the media with C/N ratios ranging from 2.6 to 2.5 and was progressively decreased for the media with C/N ratios from 2.5 to 2.3. I/S ratio proximately remained stable with C/N ratios from 2.3 to 0.1 which was an optimum point of C/N ratio (data not shown). The productions rate of extracellular inulinase and invertase per gram dry mycelium was about 12 and 30 times higher in optimum medium compared to the basal medium, respectively (data not shown). The effect of C/N ratio on enzyme production was strain dependent [35]. Carbon and nitrogen were critical nutritional parameters in the productions of intra- and extracellular inulinase and invertase and biomass built-up by *A. niger* ATCC 20611. Upon the selection of the preferred carbon and nitrogen sources, the effect of them on C/N ratios during intra- and extracellular inulinase and invertase productions was studied. So, to investigate the change of the C/N ratios, the quantity of nitrogen content (mixture of yeast extract and NaNO_3_) in the medium was varied and the quantity of carbon content (sucrose) was fixed. Based on our previous study, organic and inorganic nitrogen sources have been claimed to promote cell growth and synthesis of enzymes [13, 14]. Maximum enzymes were obtained under carbon limitation, while enough nitrogen was available for the cells. This result might be the consequence of channelling more nitrogen for cell growth and intra- and extracellular enzymes production.

In the formulation of medium, natural and relative concentrations of carbon and nitrogen sources are very important [36]. The significant relation between C/N ratio, enzymes production, biomass, and I/S ratio was observed. Clearly, the enzymes production and growth were strongly related to a low C/N ratio that resulted in better production of enzymes and biomass. Productions of intra- and extracellular enzymes were generally associated with the stationary phase of carbon, when an excess of nitrogen was channelled into secondary metabolism [37]. The imbalanced ratio between carbon and nitrogen content in the medium led to a decrease in the enzymes production. Results from this study suggested that the production of intra- and extracellular enzymes and growth were highly dependent on C/N ratios. A high productivity of enzymes was generally obtained by using a slowly metabolized nitrogen source by limiting the carbon content conditions. However, the presence of excess nitrogen under consumption of all carbon greatly enhanced the rate of production of enzymes. Apparently, the metabolic pathways for the synthesis of intra- and extracellular inulinase and invertase from nitrogen were much slower than the pathways that convert nitrogen to biomass. Therefore, carbon limitation enhanced enzymes production by diverting the extra nitrogen to enzymes production. This ability had made *A. niger* ATCC 20611 an important microorganism in industrial production of secreted glycoproteins. 

## 4. Conclusions

Results from this study have demonstrated that *A. niger* ATCC 20611 is an efficient intra- extracellular inulinase and invertase producer. Also, the ultrasonic method was found more effective than grinding by porcelain mortar to extract the intracellular enzymes. The results showed that optimization of the reaction parameters by RSM design was an effective method for attaining high intra- and extracellular inulinase and invertase productions and biomass. The C/N ratio of the culture could indicate the intra- and extracellular inulinase and invertase productions and growth. The high yields of enzymes and growth were generally obtained by using an excess nitrogen source under conditions of carbon limitation. The developed model and optimum conditions could be used for future process scale-up. 

## Figures and Tables

**Table 1:** Range and levels of experimental variables.

Factors	Level of factors		
	Low (−)	Medium (0)	High (+)
Sucrose (A/(w/v))	4.0	10	16
Yeast extract (B/(w/v))	1.0	2.5	4.0
NaNO_3 _(C/(w/v))	1.0	2.0	3.0
Zn^+2^ (D/(v/v))	1.0	1.5	2.0
Triton X-100 (E/(v/v))	0.5	1.0	1.5

**Table 2:** Extracellular inulinase and invertase productions by different commercial media.

Production media	Extracellular inulinase activity (U/mL)	Extracellular invertase activity (U/mL)
A	157 ± 0.94	536 ± 2.08
B	707 ± 3.14	1167 ± 7.05
C	697 ± 2.16	955 ± 5.02
D	2553 ± 9.17	1983 ± 8.03
E	1664 ± 6.16	1052 ± 6.05
F	880 ± 4.15	663 ± 3.08

A: yeast extract 3 g, malt extract 3 g, peptone 5 g, and dextrose 10 g.

B: potato dextrose broth.

C: for the production of inulinase [inulin (1%), NH_4_Cl (2.4%), and Mg_2_SO_4_·7H_2_O (1.2%)] and invertase [inulin (5%), NH_4_Cl (4.8%), and Mg_2_SO_4_·7H_2_O (1.2%)].

D: sucrose (20 g), yeast extract (2 g), NaNO_3_ (2 g), MgSO_4_·7H_2_O (0.05 g), and K_2_HPO_4_ (0.5 g).

E: inulin (10 g), yeast extract (1.5 g), NH_4_NO_3_ (2.3 g), (NH_4_)_2_HPO_4_ (3.7 g), KH_2_PO_4_ (1.0 g), and MgSO_4_ (0.5 g).

F: inulin (2 g), peptone (2 g), (NH_4_)H_2_PO_4_ (1.2 g), NaCl (0.5 g), and MgSO_4_·7H_2_O (0.05 g).

**Table 3:** Determination of intracellular inulinase and invertase productions by the two different methods.

Inulinase activity (U/mL)			Invertase activity (U/mL)		
Intracellular		Extracellular	Intracellular		Extracellular
SN	PM		SN	PM	
158 ± 0.80	82 ± 0.10	356 ± 1.56	295 ± 1.20	127 ± 0.30	250 ± 1.18

Mean ± SD, *P* ≤ 0.05 significant.

SN: sonication.

PM: porcelain mortar.

**Table 4:** Analysis of variance (ANOVA).

Responses	Model	Lack of fit
Extracellular inulinase (U/mL)		
*P* value	0.0002	0.1120
*F* value	10.21	3.70
*R* ^2^	0.93	—

Extracellular invertase (U/mL)		
*P* value	<0.0001	0.1536
*F* value	19.22	2.99
*R* ^2^	0.96	—

Biomass (mg/mL)		
*P* value	<0.0001	0.0601
*F* value	17.54	5.46
*R* ^2^	0.96	—

C/N%		
*P* value	<0.0001	0.1704
*F* value	18.84	2.77
*R* ^2^	0.96	—

**Table 5:** Composition of various experiments of the central composite design (CCD) for independent variables and predicted and experimental values of responses.

Run no.	Sucrose (w/v)	Yeast extract (w/v)	NaNO_3_ (w/v)	Zn^+2^ (v/v)	Triton X-100 (v/v)	Extracellular inulinase (U/mL)		Extracellular invertase (U/mL)		Biomass mg/mL		C/N	
						Actual	Predicted	Actual	Predicted	Actual	Predicted	Actual	Predicted
1	16.00	4.00	1.00	2.00	0.50	1032.23	1140.22	1318.67	1311.74	24.25	24.51	2.1	2.09
2	16.00	1.00	3.00	2.00	0.50	1430.86	1367.56	1764.30	1698.08	36.29	37.83	1.5	1.56
3	4.00	4.00	3.00	1.00	1.50	219.53	123.44	458.12	461.95	19.00	16.24	2.6	2.65
4	16.00	4.00	3.00	1.00	0.50	862.46	867.01	903.05	833.45	21.18	19.06	2.5	2.51
5	16.00	4.00	1.00	1.00	1.50	884.57	748.36	1128.66	1059.05	20.21	20.19	2.4	2.45
6	16.00	1.00	1.00	2.00	1.50	1332.80	1297.23	1395.53	1416.14	34.61	34.69	1.7	1.63
7	4.00	1.00	3.00	2.00	1.50	1235.41	1172.11	1342.99	1293.56	29.04	30.75	2.0	2.02
8	4.00	4.00	1.00	2.00	1.50	1030.99	1038.33	1293.56	1376.84	23.20	23.00	2.2	2.19
9	16.00	1.00	3.00	1.00	1.50	1402.18	1366.60	1621.43	1625.26	35.40	34.74	1.6	1.61
10	4.00	4.00	3.00	2.00	0.50	930.16	970.30	1202.20	1198.66	22.44	21.60	2.3	2.38
11	4.00	1.00	1.00	1.00	0.50	1259.39	1163.32	1377.56	1321.19	29.76	29.64	1.9	1.96
12	4.00	2.50	2.00	1.50	1.00	1282.62	1490.61	1382.73	1404.97	30.81	33.02	1.8	1.60
13	16.00	2.50	2.00	1.50	1.00	1598.38	1756.51	2068.23	2256.15	37.11	38.01	1.4	1.35
14	10.00	1.00	2.00	1.50	1.00	2167.92	2461.75	2413.59	2561.17	39.37	36.80	1.1	1.02
15	10.00	4.00	2.00	1.50	1.00	1904.32	1976.61	2352.84	2415.42	38.69	44.36	1.2	1.03
16	10.00	2.50	1.00	1.50	1.00	2247.08	2399.61	2452.98	2481.99	39.62	39.62	1.0	0.98
17	10.00	2.50	4.00	1.50	1.00	1881.45	2095.04	2306.64	2487.78	38.07	41.18	1.3	1.07
18	10.00	2.50	2.00	1.00	1.00	2259.90	2619.30	2496.12	2684.04	40.96	46.63	0.9	0.73
19	10.00	2.50	2.00	2.00	1.00	3681.21	3687.93	3854.48	3876.72	73.39	70.82	0.1	0.02
20	10.00	2.50	2.00	1.50	0.50	2287.32	2294.04	2528.36	2731.03	43.17	44.44	0.8	0.60
21	10.00	2.50	2.00	1.50	1.50	2383.92	2743.32	2677.46	2684.94	43.29	45.13	0.7	0.64
22	10.00	2.50	2.00	1.50	1.00	2726.29	2681.88	2854.61	2887.25	49.57	48.29	0.6	0.61
23	10.00	2.50	2.00	1.50	1.00	2959.09	2681.88	3079.77	2887.25	48.69	48.29	0.4	0.61
24	10.00	2.50	2.00	1.50	1.00	3166.54	2681.88	3105.58	2887.25	52.16	48.29	0.3	0.61
25	10.00	2.50	2.00	1.50	1.00	2807.25	2681.88	2939.38	2887.25	49.92	48.29	0.5	0.61
26	10.00	2.50	2.00	1.50	1.00	3214.72	2681.88	3297.57	2887.25	53.55	48.29	0.2	0.61

**Table 6:** Optimum conditions for independent variables and predicted and experimental values of responses.

Run no.	Sucrose (w/v)	Yeast extract (w/v)	NaNO_3_ (w/v)	Zn^+2^ (v/v)	Triton X-100 (v/v)	Extracellular inulinase (U/mL)		Extracellular invertase (U/mL)		Biomass mg/mL		C/N	
						Actual	Predicted	Actual	Predicted	Actual	Predicted	Actual	Predicted
2	7.17	1.47	1.28	2.00	0.50	3802.43	3733.14	3858.98	3862.30	72.54	73.49	0.02	0.00
3	7.35	1.73	1.09	1.99	0.50	3754.98	3812.80	3930.42	3917.98	72.98	73.39	0.15	0.10
4	7.13	1.76	1.42	2.00	0.50	3793.65	3765.34	3906.97	3885.80	73.03	73.48	0.06	0.08
5	8.95	2.16	1.00	2.00	0.50	3698.96	3768.61	3865.38	3866.43	74.07	73.47	0.02	0.03
